# The Association Between Antibiotic Use and the Incidence of Third-Generation Cephalosporin-Resistance in *Escherichia coli* Bloodstream Infections: An Ecological Study

**DOI:** 10.3390/antibiotics15020187

**Published:** 2026-02-09

**Authors:** Adi Cohen, Elizabeth Temkin, Mitchell J. Schwaber, Yehuda Carmeli, BSI Working Group

**Affiliations:** 1 National Institute for Antibiotic Resistance and Infection Control, Israel Ministry of Health, Tel Aviv 6423906, Israel; adicoh@tlvmc.gov.il (A.C.); lizt@tlvmc.gov.il (E.T.); mitchells@tlvmc.gov.il (M.J.S.); 2 Gray Faculty of Medical and Health Sciences, Tel Aviv University, Tel Aviv 6997801, Israel

**Keywords:** *Escherichia coli*, bloodstream infection, antibiotic resistance, antibiotic consumption, COVID-19, interrupted time series analysis

## Abstract

**Background/Objectives**: The COVID-19 period was marked by changes in antibiotic use and in the incidence of bacterial infections. We examined the association between antibiotic use and the proportion of *Escherichia coli* bloodstream infections (BSI) that were third-generation cephalosporin-resistant (3GC-R), using the COVID-19 period as a natural experiment. **Methods**: Data for this ecological study came from Israeli national surveillance systems for BSI and antibiotic consumption in 2015–2023. We performed interrupted time series analyses with a 1-year lag to examine the impact of COVID-19 on the proportion of *E. coli* BSI that were 3GC-R. We used linear regression to test the association between antibiotic use and 3GC resistance. **Results**: The majority of national antibiotic use was in outpatient settings; it was stable between 2015–2019, dropped by 19.4% in 2020, then increased gradually, but in 2023 remained 10.8% lower than before the pandemic. Incidence of *E. coli* BSI per 100,000 population increased from 62.6 in 2015 to a peak of 66.0 in 2019, with a small, non-significant change in the proportion of *E. coli* BSI that were 3GC-R (0.339 in 2015 vs. 0.335 in 2020). In 2020, the incidence of both 3GC-susceptible and 3GC-R *E. coli* BSI decreased. In 2021, only 3GC-R BSI declined, resulting in the proportion resistant dropping significantly by 0.05 (95% CI: 0.03–0.07). Post-pandemic, BSI incidence rose but remained below the 2019 rate. The proportion resistant after 2021 rose by 0.02 per year relative to the pre-COVID slope (95% CI: 0.02–0.03), such that it was higher in 2023 (0.341) than in 2019 and 2015. There was a significant positive linear relationship between antibiotic use and resistance: the proportion of *E. coli* BSI that were 3GC-R increased by 0.02 for each increase of one defined daily dose of antibiotic per person (95% CI: 0.001–0.03). **Conclusions**: Reduced outpatient antibiotic use during COVID-19 was followed by a reduction in the proportion of *E. coli* BSI that were 3GC-R.

## 1. Introduction

*Escherichia coli* is an important cause of morbidity and mortality worldwide. *E. coli* ranked second among 85 pathogens responsible for disability-adjusted life-years (DALYs) in high-income countries in 2019 [1]. Antibiotic resistance increases the fatality of *E. coli* infections [2], and resistance among *E*. *coli* is rising. Over the last two decades, third-generation cephalosporin (3GC) resistance among *E. coli* has reached high levels in most countries. In Italy, for example, the percentage of *E. coli* invasive isolates resistant to 3GC rose from 3% in 2002 to 28% in 2024; in 2024, no European country had less than 6% 3GC resistance among *E. coli* invasive isolates [3]. Globally, in 2021, 33,100 deaths were estimated to be attributable to infections caused by 3GC-resistant (3GC-R) *E. coli* (i.e., the deaths would not have occurred if the infections had been antibiotic-susceptible) [4]. 3GC resistance is primarily driven by extended-spectrum beta-lactamase (ESBL) production and often co-occurs with resistance to other antibiotic classes, leading to a multidrug resistance (MDR) phenotype.

Antibiotic use is tightly linked to antibiotic resistance [5]. A study of an intervention that reduced the prescribing of quinolones in the community found a corresponding decline in quinolone resistance in outpatient urinary *E. coli* isolates [6]. However, the association between antibiotic consumption and resistance is not consistent across antibiotic classes. One study of changes in outpatient antibiotic prescribing and resistance in urinary *E. coli* reported parallel effects (e.g., decreased amoxicillin use and resistance), opposite effects (e.g., decreased cefalexin use and increased resistance), and cross-agent effects (e.g., higher nitrofurantoin use and decreased resistance to trimethoprim) [7]. An intervention in Scotland to reduce outpatient prescribing of fluoroquinolones, cephalosporins, and co-amoxiclav found modest, delayed effects on resistance among coliforms causing community-acquired bloodstream infection (BSI); for co-amoxiclav, the reduction in resistance was not significant [8].

In Israel, 3GC resistance among *E. coli* is longstanding: in 2001, 9% of *E. coli* blood isolates were 3GC-R, compared to a median of 1% among European countries [9]. By 2023, according to Israel’s national antibiogram (which includes the first blood isolate per patient), 32% of *E. coli* were 3GC-R [10], while the European median had risen to 14% [3]. Regarding antibiotic consumption in the community, which comprises 80–95% of antibiotic use in humans worldwide [11], Israel falls into the middle third when compared to European countries [12]. Israel has set a national goal to drop to the lower third and to shift from antibiotics in the Watch category to the AWaRe category [13] as much as possible. There is a lack of data from Israel about the influence of antibiotic use on 3GC resistance among *E. coli*. The COVID-19 pandemic provides an opportunity to study the association between antibiotic use and the incidence and proportion of antibiotic-resistant infections, as it was a period characterized by changes in both antibiotic consumption and infection incidence. For example, in the US, outpatient antibiotic use in 2020 was significantly lower than in 2019 because of decreased use of outpatient services and the beneficial impact of masking and distancing on transmission of respiratory illnesses; in 2021, antibiotic use rebounded to above 2019 levels [14]. The rate of hospital-acquired infections (HAIs), including BSI, rose during the pandemic [15,16]. The CDC reported an increase of at least 15% in the incidence of antibiotic-resistant HAIs (78% for carbapenem-resistant *Acinetobacter*) [14]. In contrast, a meta-analysis of 23 studies of the incidence or proportion of resistant infections during the pandemic found no change for Gram-positive bacteria and a non-significant increase for Gram-negative bacteria [17].

In this study, we examined the association between antibiotic use and the proportion of *E. coli* BSI that were 3GC-R. We analyzed the incidence and 3GC resistance patterns of *E. coli* BSI and levels of antibiotic use in Israel in 2015–2023 and used the COVID-19 period as a natural experiment. We hypothesized that a decrease in antibiotic use during the COVID-19 period would lead to a decrease in the proportion of *E. coli* BSI that were 3GC-R.

## 2. Results

### 2.1. Antibiotic Use

**Figure 1:**
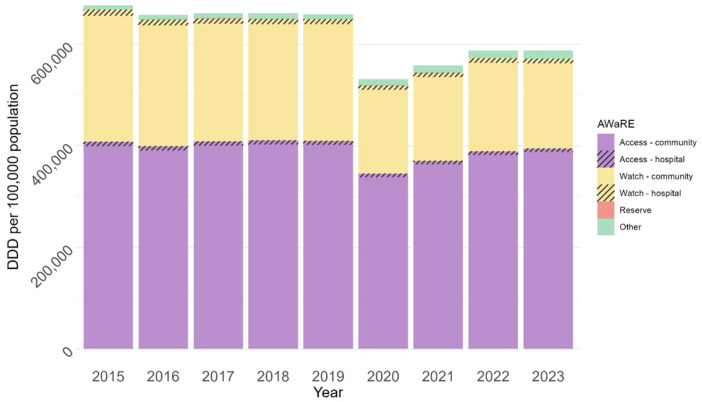
Annual antibiotic consumption by AWaRe category and site, Israel, 2015–2023. Antibiotics in the Reserve category comprised < 600 DDD per 100,000 population each year.

Figure 1 shows antibiotic use by AWaRe category in 2015–2023. There was little change in use from 2015 to 2019 and then a 19.41% drop in 2020 (95% CI: 19.38–19.44%, *p* < 0.001), the first year of COVID-19. In the following 3 years, antibiotic use rose, but in 2023, it remained 10.8% lower than before the pandemic (95% CI: 10.8–10.9%, *p* < 0.001). Antibiotic use in hospitals (in monitored wards, which account for about half of all inpatient antibiotic use) comprised less than 3% of all use.

### 2.2. BSI Incidence and Resistance

There were 51,829 events of *E. coli* BSI in 46,446 patients between 2015 and 2023. Data on 3GC susceptibility were not available for 455 (0.9%) events; they were excluded from further analysis. Of the 51,374 remaining events, 34,375 (66.9%) of cases were caused by 3GC-susceptible (3GC-S) *E. coli* and 16,999 (33.1%) by 3GC-R *E. coli*. BSI were community-onset (CO) in 42,469 (82.7%) events and hospital-onset (HO) in 8905 (17.3%) events.

**Figure 2:**
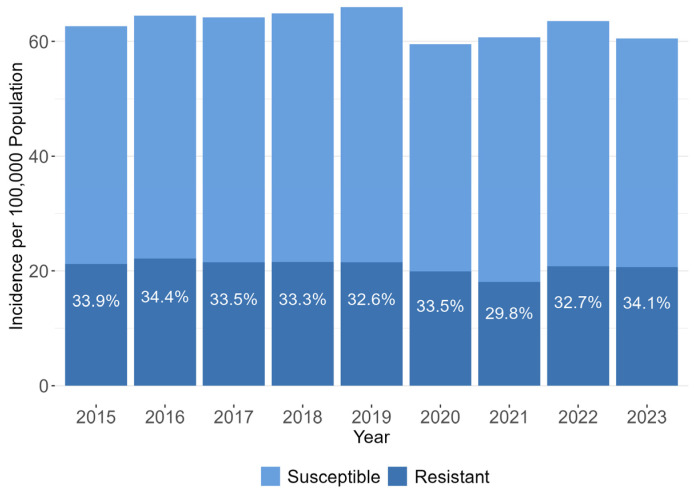
Annual incidence of *E. coli* BSI, by third-generation cephalosporin susceptibility, Israel, 2015–2023. Labels indicate the percentage resistant.

Figure 2 shows the observed incidence trends in *E. coli* BSI per 100,000 population during the study period. The incidence increased by 5.4% from 62.6 in 2015 to a peak of 66.0 in 2019, driven mostly by a rise in 3GC-S cases, with only a minor change in the incidence of 3GC-R cases. In 2020, there was a decrease in the incidence of both 3GC-S and 3GC-R *E. coli* BSI, for a total incidence of 59.9. In 2021, only 3GC-R BSI continued to decline (total incidence 60.7). After the peak years of the pandemic, the incidence of *E. coli* BSI began to rise, but remained below the 2019 rate.

**Figure 3:**
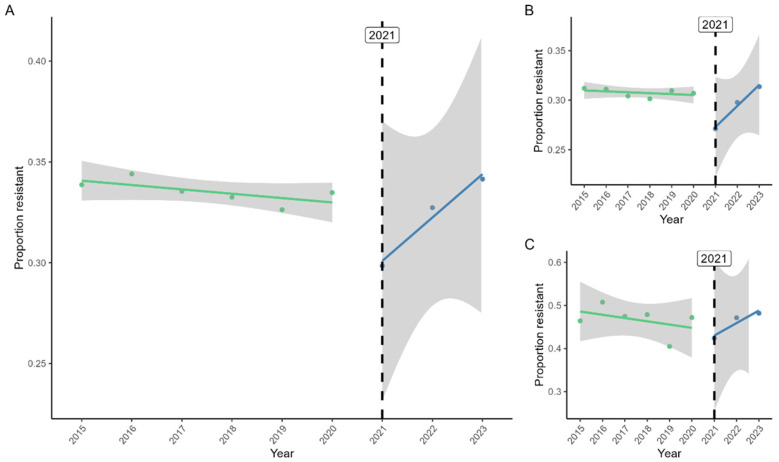
Interrupted time series analysis of the proportion of *E. coli* BSI that were third-generation cephalosporin-resistant. (**A**) all BSI, (**B**) community-onset BSI, (**C**) hospital-onset BSI. Note that for visibility, the scale of the y-axis differs between graphs. Shaded areas are 95% confidence intervals.

Figure 3 presents the interrupted time series analysis of the proportion of *E. coli* BSI that were 3GC-R. For all BSI (Figure 3A), this proportion decreased slightly but not significantly between 2015 and 2020. The proportion dropped significantly from 2020 to 2021 by 0.05 (95% CI: 0.03–0.07, *p* < 0.001). Following this drop, resistance increased by 0.02 per year relative to the pre-COVID slope (95% CI: 0.02–0.03, *p* < 0.001), such that the proportion resistant in 2023 was higher than in 2015. Figure 3B shows only CO *E. coli* BSI, which mirrored the pattern for all BSI. Among HO *E. coli* BSI (Figure 3C), there was no significant drop in resistance between 2020 and 2021, but the increase in the following years was still evident (slope: 0.04, 95% CI: 0.01–0.06, *p* = 0.02).

### 2.3. Association Between Antibiotic Use and 3GC Resistance

**Figure 4:**
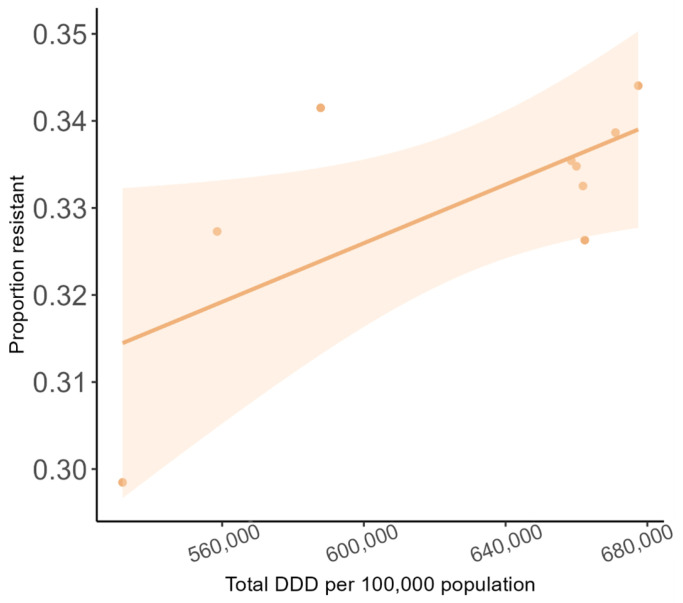
Association between antibiotic use and the proportion of *E. coli* BSI that were third- generation cephalosporin-resistant. Each point represents a year. Shaded area is 95% confidence interval.

Figure 4 shows the association between total antibiotic use and the proportion of all *E. coli* BSI that were 3GC-R (in the following year). There was a significant positive linear relationship: the proportion of *E. coli* BSI that were 3GC-R increased by 0.02 for each increase of 1 DDD per person (95% CI: 0.001–0.03, *p* = 0.04). The results were similar when examining antibiotic use in the community and CO BSI. We did not examine this association for HO BSI, as hospital antibiotic use was a small proportion of all antibiotic use, and our measure of inpatient antibiotic use was incomplete.

In multivariable analyses of defined daily doses (DDD) of specific antibiotic classes (first- and second-generation cephalosporins, 3GC, beta-lactamases, fluoroquinolones and other), we detected no significant associations between any class and the proportion of *E. coli* BSI that were 3GC-R in the following year. Likewise, in multivariable analyses of DDD by AWaRe categories, we detected no significant associations between any category and the proportion of 3GC resistance in the following year.

## 3. Discussion

We used country-level data from Israel to study the association between antibiotic consumption and the proportion of *E. coli* BSI that was 3GC-R in 2015–2023. This time span included the COVID-19 pandemic, which affected both antibiotic use and bacterial infection incidence. We found that antibiotic use (the vast majority [97%] of which was prescribed in the community) dropped sharply (19%) in the first year of the pandemic and, while rising since 2021, remained lower in 2023 than in the pre-pandemic period. Consistent with our hypothesis that a decrease in antibiotic use during the COVID-19 period would lead to a decrease in 3GC resistance, we observed that the decrease in outpatient antibiotic use in 2020 was followed a year later by a significant 5% drop in the proportion of *E. coli* BSI that were 3GC-R. We found a significant positive association between total antibiotic use and the proportion of *E. coli* BSI that were 3GC-R. We found no such association for specific antibiotic classes or AWaRe categories.

There are several ways to measure antibiotic resistance. Measuring the proportion of organisms that are resistant is useful for clinicians choosing empiric antibiotic treatment but may not reflect the burden of resistance; burden is best measured as an incidence rate and is useful for public health professionals [18]. In this study, we chose to focus on the former. The relationship between these two measures is complex. Burton et al. pointed out that they do not necessarily mirror each other. In their study of methicillin-resistant *Staphylococcus aureus* (MRSA) central line-associated bloodstream infections (CLABSIs) in US intensive care units over 10 years, they observed that the proportion of *S. aureus* CLABSIs that were MRSA increased by 26%, while the incidence of MRSA CLABSIs had decreased by half; what appeared to be a setback (higher resistance) was actually an improvement (fewer infections) [19]. This phenomenon was also evident in our study between 2019 and 2020, when the percentage of *E. coli* BSI that were 3GC-R rose while the incidence of 3GC-R *E. coli* BSI decreased. However, we also observed other patterns: between 2015 and 2016, the percentage resistance and incidence of 3GC-R BSI rose in tandem; between 2022 and 2023, the percentage resistance increased, while the incidence of 3GC-R *E. coli* BSI remained stable.

Antibiotic use can increase the incidence of resistant infections by facilitating transmission and by promoting the progression from carriage of resistant organisms to infection. Antibiotic treatment facilitates the transmission of resistant bacteria in three ways: (1) By disrupting the normal flora, antibiotics increase the vulnerability of non-carriers of resistant bacteria to become colonized by a resistant strain. (2) By successfully eradicating susceptible strains in a host, antibiotics reduce transmission of susceptible strains, making other hosts more likely to acquire resistant strains. (3) By enabling overgrowth of resistant bacteria among carriers, antibiotics increase carriers’ contagiousness [20]. In addition, this overgrowth of resistant bacteria (higher bacterial load) among carriers increases their risk of progressing from the carrier state to clinical infection [20,21], thereby increasing the incidence of resistant infections. In our study, the percentage of 3GC resistance among *E. coli* blood isolates in 2021 was the lowest since 2015. Previous studies reporting a similar pattern concluded that the decrease in antibiotic resistance among *E. coli* during the pandemic resulted from restrictions that reduced contact between people (and thus transmission of resistant organisms), rather than reduced antibiotic use. In Australia, after lockdown restrictions on entrance to the country were lifted, 3GC resistance among *E. coli* urine isolates rose even though antibiotic use did not, indicating that increased human interaction drove the change in resistance [22]. Likewise, Stanley et al. concluded that, in South West England, the drop in resistance to piperacillin-tazobactam and ciprofloxacin among *E. coli* blood isolates in 2020 was too abrupt to be explained by a drop in antibiotic use, whose effect would be expected to be seen at a lag of up to 6 months [23]. In our study, 3GC resistance was lowest in 2021, while antibiotic use was lowest in 2020, illustrating this lag between antibiotic consumption and resistance. If decreased human contact were the main cause of decreased resistance in Israel, we would expect to see a lower percentage of resistance in 2020 (not 2021), which had more lockdown days (68) than in 2021 (37 days) and more restrictions on international travel [24]. Social distancing may have had a smaller impact on the transmission of resistant bacteria in Israel because of higher resistance levels. The percentage of 3GC resistance among *E. coli* blood isolates in 2020 was 12% in Australia [25] and 11% in South West England [26], compared to 34% in Israel. Thus, the probability of interacting with a 3GC-R *E. coli* carrier in Israel was high, even when opportunities for interaction were fewer. Moreover, there were no restrictions on interactions within households, which may play an important role in community transmission [27]. The role of household transmission may be more influential on the total number of colonized persons in regions with a high prevalence of carriers of resistant bacteria (such as Israel) than in lower-prevalence areas such as northern Europe, where travel may play a more important role.

We found that 3GC resistance was associated with total antibiotic use, but not the use of specific antibiotic classes. Previous patient-level studies have reported a positive association between infection by 3GC-R (or ESBL-producing) Enterobacterales and exposure to 3GC [28,29], second-generation cephalosporins [30], fluoroquinolones [31] and trimethoprim-sulfamethoxazole [32]. Likewise, studies have found a greater risk of colonization or infection by ESBL-producing Enterobacterales among patients exposed to antibiotics in any AWaRe category, but the risk was higher for those in the Watch or Reserve categories [33]. We suspect that associations with specific antibiotic classes and AWaRe categories were not significant in our ecological study because of the relatively small number of years modeled.

Our study has several limitations. As just noted, we had only 9 years’ worth of data, limiting statistical power. Second, we used annual data on antibiotic use and BSI incidence; our results may have been more precise if we had used shorter units of analysis. Third, our measurement of inpatient antibiotic use did not cover all wards; however, 48.3% of hospital-onset BSI were in ward types that are included in antibiotic use surveillance, and the great majority of antibiotic use was in the community.

## 4. Materials and Methods

### 4.1. Study Design and Data Sources

We conducted an ecological study. The sample consisted of all patients in Israel with *E. coli* BSI detected in 2015–2023. Data on *E. coli* BSI came from the BSI surveillance system managed by the National Center for Infection Control (NCIC) in the Israeli Ministry of Health. As few blood cultures are performed in laboratories that did not report to the system during the study period, the system captures 97% of all *E. coli* BSI. Data on antibiotics came from the NCIC’s antibiotic surveillance system, to which all hospitals and health maintenance organizations report annual antibiotic use. This surveillance system measures antibiotic consumption using the Defined Daily Dose (DDD) method (and using, for all years, the DDD values revised in 2019) [34], as described previously [12]. In hospitals, surveillance is limited to internal medicine wards, general surgery wards, and general intensive care units, which account for about half of all inpatient antibiotic use. Hospitalization days were available from the Ministry of Health. Data on annual population size were taken from the Israel Central Bureau of Statistics [35].

### 4.2. Definitions

We defined a BSI event as *E. coli* isolated in a blood culture. A repeat positive blood culture after 14 days was considered a new event, according to the definition used by the National Healthcare Safety Network [36]. An isolate was classified as 3GC-R if it was resistant or intermediately susceptible to ceftazidime or ceftriaxone. For each BSI event, we classified resistance based on the first positive culture. BSI was defined as community-onset (CO) if the positive blood culture was taken in the first 3 days of hospitalization and as hospital-onset (HO) if taken after day 3. The COVID-19 period was defined as starting in 2020. Although *E. coli* BSI incidence varies by season [37], our unit of time was years because antibiotic data were only reported annually.

### 4.3. Laboratory Methods

All blood cultures were processed by microbiology laboratories that use automated blood culture systems for species identification and antibiotic susceptibility testing [36].

### 4.4. Statistical Analysis

To calculate the annual BSI incidence rate, the denominator for total BSI and CO BSI was the Israeli population, and the denominator for HO BSI was patient-days. To calculate annual antibiotic use, the numerator was DDDs and the denominator was the Israeli population.

Hospitalization date was reported to the BSI surveillance system beginning in 2018, allowing for classification of BSI as CO or HO. For the years 2015–2017 (and for rare cases of missing data in later years), we imputed the proportion of BSIs that were CO or HO using the average proportions of CO or HO in 2018–2023, stratified by 3GC resistance status.

We calculated rate ratios and their 95% exact confidence intervals (CI) to compare antibiotic use between years. We performed an interrupted time series analysis (ITSA) to examine the impact of COVID-19 on the proportion of *E. coli* BSI that are 3GC-R, using a Newey-West standard error correction to account for autocorrelation. We performed ITSAs for all *E. coli* BSI and separately for CO and HO BSI. We chose a priori to assume a one-year lag between antibiotic use and antibiotic resistance; a previous study used a 6-month lag [8], but, as noted above, our unit of time was years. We used linear regression to test the association between antibiotic use and the proportion of *E. coli* BSI that were 3GC-R, also with a one-year lag. We examined total antibiotic use, specific antibiotic classes, and the World Health Organization’s AWaRe categories (Access, Watch, Reserve and other) [13].

All analyses were done in R Studio/2023.06.0+421.

## 5. Conclusions

In conclusion, the natural experiment of reduced outpatient antibiotic use during COVID-19 was followed by a reduction in the proportion of *E. coli* BSI that were 3GC-R in Israel. Both antibiotic use and *E. coli* BSI incidence have risen since the peak of COVID-19 but remain lower than in the years before the pandemic. Our findings support efforts to decrease total antibiotic use in the community in order to reduce the proportion of *E. coli* that are 3GC-R.

## Data Availability

Annual reports on antibiotic use are available (in Hebrew) at the National Center for Infection Control’s website: https://www.gov.il/he/Departments/DynamicCollectors/infection-control-reports?skip=0&infection_control_reports_subject=2 (accessed on 4 February 2026). Patient-level data on BSI come from a governmental database with restricted access. Bi-monthly and annual reports on *E. coli* BSI incidence are available (in Hebrew) at https://www.gov.il/he/Departments/DynamicCollectors/infection-control-reports?skip=0&infection_control_reports_subject=4 (accessed on 4 February 2026).

## Abbreviations

The following abbreviations are used in this manuscript:

3GC	Third-generation cephalosporin
3GC-R	Third-generation cephalosporin-resistant
3GC-S	Third-generation cephalosporin-susceptible
BSI	Bloodstream infection
CO	Community-onset
HO	Hospital-onset
DDD	Defined daily dose
ITSA	Interrupted time series analysis
NCIC	National Center for Infection Control (NCIC)
HAI	Hospital-acquired infection
ESBL	Extended-spectrum beta-lactamase
